# Circular RNA expression profile of systemic lupus erythematosus and its clinical significance as a potential novel biomarker

**DOI:** 10.1007/s13258-022-01315-z

**Published:** 2022-09-27

**Authors:** Wenyu Li, Runge Fan, Cheng Zhou, Yue Wei, Shunsheng Lin, Sijian Wen, Wen Zeng, Wei Hou, Cheng Zhao, Youkun Lin

**Affiliations:** 1 Department of Dermatology/Venerology, the First Affiliated Hospital of Guangxi Medical University, 530021 Nanning, China; 2 Department of Rheumatology, the First Affiliated Hospital of Guangxi Medical University, 530021 Nanning, China; 3 Key Laboratory of Thalassemia MedicineGuangxi Key Laboratory of Thalassemia Research, Chinese Academy of Medical Sciences, Nanning,, China

**Keywords:** Systemic lupus erythematosus (SLE), circRNAs, RNA sequencing, hsa_circ_0006689

## Abstract

**Background:**

Circular RNAs (circRNAs) are a class of endogenous noncoding RNAs that are more abundant, specific, and highly organized than linear RNAs. Increasing evidence supports that circRNAs may serve as diagnostic biomarkers in many diseases, but their potential as biomarkers in systemic lupus erythematosus (SLE) remains unclear.

**Objective:**

We investigated the critical circRNAs involved in SLE progression and explored their potential application as biomarkers in SLE.

**Method:**

RNA sequencing was conducted on peripheral blood mononuclear cells (PBMCs) from 4 SLE patients and 4 healthy volunteers. CircRNA profile data were analyzed to identify differentially expressed circRNAs and visualized via R software. After screening, qPCR analysis of target circRNA expression was performed using PBMCs from 31 SLE patients and 35 healthy volunteers. Correlations between circRNA expression levels and the SLEDAI score were assessed via Spearman correlation analysis. Finally, the performance of circRNAs as biomarkers in SLE was examined by receiver operating characteristic curve analysis.

**Results:**

The result identified six differentially expressed circRNAs between SLE patients and healthy controls: hsa_circ_0006689, hsa_circ_0070562, hsa_circ_0006117, hsa_circ_0007683, hsa_circ_0042519, and hsa_circ_0008647. The validation analysis showed differing relative expression levels of hsa_circ_0007683, hsa_circ_0042519, hsa_circ_0008647, and hsa_circ_0006689 between SLE patients and healthy volunteers (P < 0.05), and hsa_circ_0006689 expression in PBMCs correlated with the SLEDAI score (P < 0.05). Furthermore, addition of hsa_circ_0006689 expression increased the sensitivities of anti-dsDNA antibody and anti-Sm antibody levels for SLE diagnosis (from 29.03 to 61.30% and 32.26–71.00%, respectively).

**Conclusion:**

Our results suggest hsa_circ_0006689 may be a useful circRNA biomarker for SLE diagnosis and prognosis.

## Introduction

Systemic lupus erythematosus (SLE) is an autoimmune disease characterized by the widespread deposition of immune complexes. Clinically, SLE patients present with injury to various organ systems and multiple positive autoantibodies to self-antigens. SLE will eventually lead to irreversible damage to organs and even death if not treated and management early enough or appropriately (Durcan et al. 2019). The prevalence of SLE varies greatly geographically, and the prevalence of SLE in China is 40/100,000 (Chang 1983). With progress that has been made in the diagnosis and treatment of SLE in recent years, the 5-year survival rate of SLE patients has improved significantly, from 50 to 60% in the 1950s to over 90% in the 1990s (Tektonidou et al. 2017). However, SLE remains associated with a poor prognosis and compromised quality of life.

Early diagnosis and treatment are of great significance for improving the response rate and prognosis among SLE patients. In clinical practice, anti-double stranded DNA (anti-dsDNA) antibodies, anti-nuclear antibodies (ANAs), anti-nucleosome antibody (ANuA), and anti-Sm antibody are the most commonly used biomarkers for the diagnosis of SLE (Petri et al. 2012). The most commonly used classification criteria for SLE were developed by the European League Against Rheumatism (EULAR), the American College of Rheumatology (ACR), and the Systemic Lupus International Collaborating Clinics (SLICC) ([2020 Chinese guidelines for the diagnosis and treatment of systemic lupus erythematosus] 2020). However, due to the complex, diverse and heterogeneous clinical manifestations of SLE, simple, rapid and accurate diagnostic methods for SLE have yet to developed. Thus, early and accurate detection and diagnosis of SLE remains a major clinical challenge. Accordingly, new potential markers of SLE are urgently needed. The advancement of molecular biology technologies has facilitated more research into novel diagnostic markers of SLE, such as serum Galectine-9 and non-coding RNAs (e.g., microRNAs [miRNAs], long non-coding RNAs [lncRNAs]), which have shown some value in the diagnosis of SLE (Li et al. 2020a; Matsuoka et al. 2020).

Circular RNAs (circRNAs) are a type of non-coding RNA that was first reported when 80 circRNAs were identified via RNA sequencing (RNA-Seq) in 2012 and are widely present in humans, animals and plants (Salzman et al. 2012). With the characteristics of abundant expression and stable and conserved structures, circRNAs offer great potential as biomarkers for the diagnosis, monitoring, and prognosis of diseases (Han et al. 2018). In the last decades, with the development of high-throughput sequencing technology, accumulating evidence indicates that circRNAs play important roles in the development and progression of tumors, atherosclerotic vascular disease, Alzheimer’s disease, and autoimmune diseases (Altesha et al. 2019; Li et al. 2020b, c). CircRNAs have also been reported in SLE. Recently, next-generation sequencing (NGS) was applied to comprehensively analyze circRNA expression profiles in peripheral blood mononuclear cells (PBMCs) of 10 SLE patients in comparison with 10 healthy controls, and hsa_circ_0000479 was found to be a good diagnostic marker of SLE (Guo et al. 2019). In addition, one study explored the circRNA–miRNA–mRNA regulatory network for SLE by analyzing the Gene Expression Omnibus (GEO) database GSE84655, and their results provided the possible mechanism of circRNA function in SLE (Zhang et al. 2021). These studies imply that circRNAs have critical functions in SLE and thus may serve as biomarkers for the diagnosis and prognosis of SLE.

Building upon these early studies of circRNAs in SLE, further research is needed to determine the clinical value of circRNAs in SLE patients. In this study, we further analyzed the differential expression of circRNAs in PBMCs from 4 SLE patients and 4 healthy controls using high-throughput sequencing technology and then further verified and evaluated the differential expression of the identified circRNAs in 31 SLE patients through qPCR to investigate the potential of circRNAs as biomarkers for SLE. From our results, we report a new circRNA derived from *SLC15A4*, which has been previously confirmed to be susceptibility gene for SLE by a genome-wide association study (GWAS) in China, as a potential biomarker for SLE diagnosis and prognosis.

## Materials and methods

### Study participants

In the screening phase, 4 SLE patients and 4 healthy volunteers provided PMBC samples for high-throughput sequencing to screen differentially expressed circRNAs. During the validation phase, 31 SLE patients and 35 healthy volunteers were enrolled, and PBMCs from these patients were used for qPCR analysis of circRNA expression.

All SLE patients met the diagnostic criteria for SLE established by the ACR in 1997, and any patients with severe infection, pregnancy, malignancy, diabetes, or other autoimmune diseases were excluded. Demographic characteristics of age, sex and others were collected. Data for clinical parameters were collected, and clinical symptoms were recorded. The Systemic Lupus Erythematosus Activity Index (SLEDAI) score was determined for each patient. Clinical test results for anti-dsDNA antibodies, ANAs, and anti-Sm antibody were collected. All results and scores were interpreted by two experience clinicals according to established guidelines.

This study was approved by the Medical Ethics Committee of the First Affiliated Hospital of Guangxi Medical University. All study participants provided signed informed consent.

### Cell isolation and RNA extraction

Five milliliter venous blood samples were collected from each participant in early morning after fasting using EDTA anticoagulant tubes. PBMCs were separated by density gradient centrifugation following the manual for human PBMC separation solution (Solarbio life science, Beijing, China) within 4 h after sample collection. After PBMC isolation, TriZol Reagent (Invitrogen Life Technologies, Grand Island, NY, USA) was added, and total RNA was extracted using the Axygen Total RNA Extraction Kit (Axygen Scientific, Inc, Union City, CA, USA) according to the kit instructions. Briefly, cells were collected in a 1.5-ml tube, and buffer was added to digest proteins and DNA. After a series of column filtration steps, RNA was eluted with elution buffer. The whole operational process and instrument were RNAase-free.

### High-throughput RNA-Seq

Following RNA extraction, RNAs were digested with Rnase R (Thermo Fisher Scientific Inc, Waltham, MA, USA) for enrichment of circRNAs and removal of linear RNAs. After the construction of next-generation sequencing (NGS) libraries, high-throughput sequencing analysis was performed on an Illumina HiSeq™ 2500 (Gene Denovo Biotechnology Co., Guangzhou, China).

### Screening of differentially expressed circRNAs

After RNA-Seq, the STAR and DCC software programs were used to identify circRNAs. Then an analysis matrix was generated and normalized through R software (Lucent Technologies, USA). Log2 fold changes in circRNA expression were transformed. A volcano plot and heatmap comparing the two groups were generated via R software and R packages. circRNAs that showed differential expression with a P-value < 0.05 and log2 fold change > 2 were presented in the volcano plot and heatmap.

### Functional enrichment analysis

Differentially expressed circRNAs were analyzed by Gene Ontology (GO) analysis and Kyoto Encyclopedia of Genes and Genomes (KEGG) database analysis using R software. GO analysis included Biological Process (BP), Cellular Component (CC), and Molecular Function (MF) analysis. Pathway analysis was performed to classify and annotate the differentially expressed genes according to known pathway information in the KEGG database, and to investigate the relationships between gene expression differences and functional pathway changes.

### Quantitative real-time PCR (qRT-PCR)

The differential expression of circRNAs identified in the screening analysis was validated via qRT-PCR. After assessment of the total RNA quality and quantity using the Nanodrop One (Thermo Fisher Scientific Inc., Waltham, MA, USA), cDNA was synthesized on a T100 Thermal Cycler PCR machine with the PrimeScript RT Reagent Kit (Takara Biomedical Technology, Shiga, Japan) according to the manufacturer’s protocol. Then qRT-PCR was performed with TB Green qPCR Master Mix (Takara Biomedical Technology) on a Mx3000P QPCR System (Agilent Technologies, Santa Clara, CA, USA) following the manufacturer’s instructions and using the primers listed in Table 1. β-actin was used as an internal control. Three replicates were performed for each sample.

**Table 1:** Primers used for qRT-PCR.

Name	Sequence (5’- 3’)
hsa_circ_0007683	F: ACCTAGGCTATGCGAGACTC R: GCCTGAAGTCCCCAAGTACC
hsa_circ_0042519	F: GATGTTGGTCTTTGATCCAATTTG R: ATTGCTGTAATGACGGCTGC
hsa_circ_0008647	F: GACAGGGAGCCCAGAAGGTTT R: TACTGGGAAGACAGGAGGCA
hsa_circ_0006689	F: TCGGCCTTTGCTGCAGGTTAA R: CCACCTAACGACAGGATCGC
hsa_circ_0070562	F: CCCATCTTGCAGATGTGTAGAATTC R: CTGTCTGGCAAATGGGAGGT
hsa_circ_0006117	F: AGAAACTTTCCCTCCTTCAGATAAG R: ACTGGTTCTGCCGTTGATGA
β-actin	F: GTGGCCGAGGACTTTGATTG R: CCTGTAACAACGCATCTCATATT

### Statistical analysis

Data analysis and graph preparation were carried out using SPSS 22.0 (IBM Corp., Armonk, NY, USA), MedCal 19.1 software (MedCalc Software Ltd, Belgium) and GraphPad prism 8.0 software (GraphPad Software, San Diego, CA, USA). Receiver operating characteristic (ROC) curve analysis was performed using MedCal 19.1 software. The experimental data were tested for normality first, and the measurement data conforming to a normal distribution were expressed as mean ± standard deviation (x ± s). Measurement data that did not conform to a normal distribution are represented as median and quartiles (P25, P75). The t test was used to compare the means of two samples for normally distributed data; otherwise, the Mann–Whitney test was used. Spearman correlation analysis was applied to identify correlations between circRNA expression and clinical indicators. The diagnostic value of circRNAs was evaluated by ROC curve analysis. Values of P < 0.05 indicated statistical significance.

## Results

### Identification of circRNA expression profile in SLE

**Fig. 1:**
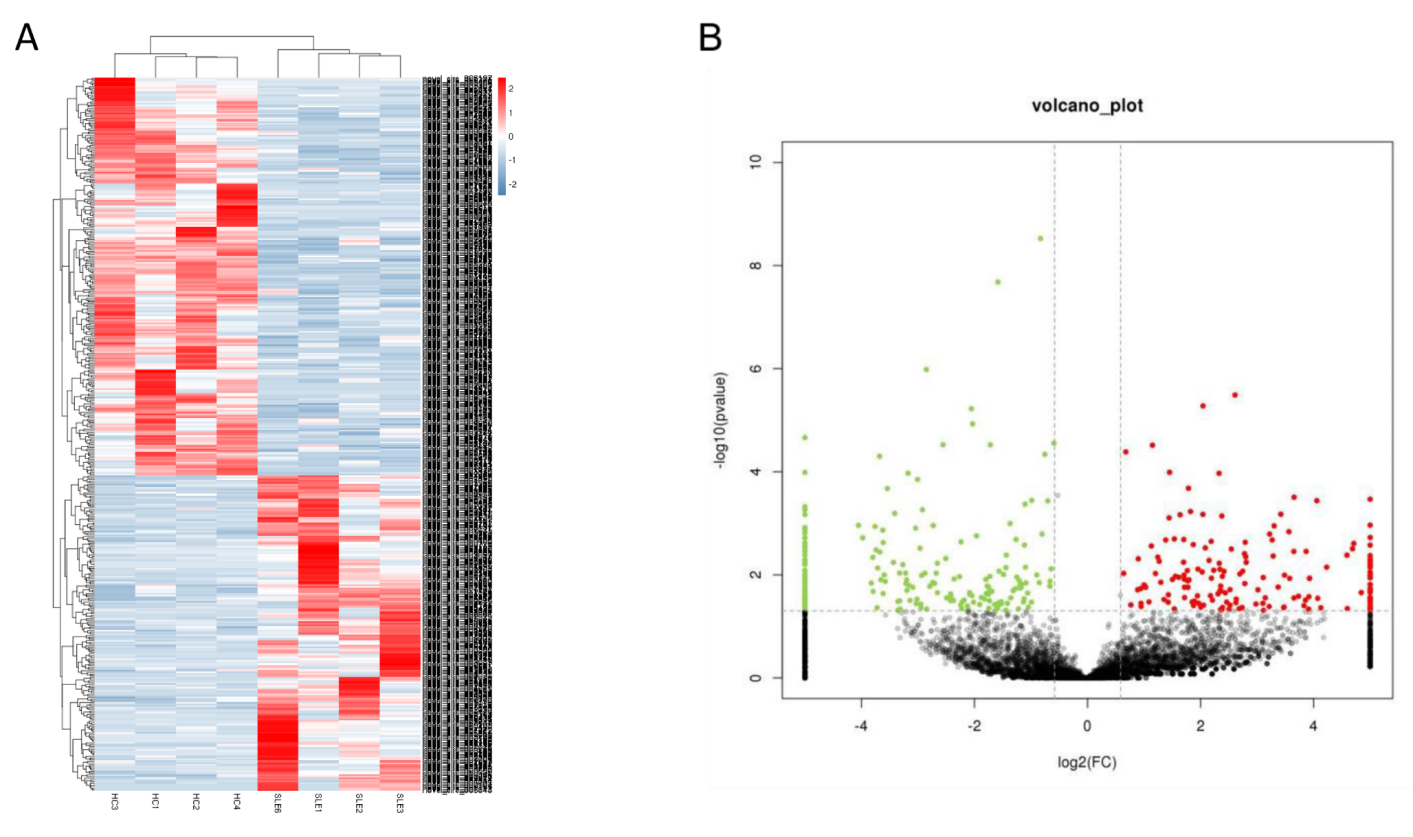
Volcano plot and heatmap of differentially expressed circRNAs between 4 SLE patients and 4 healthy volunteers (A) Heatmap analysis showed distinguishable circRNA expression profiles between the two groups. (B)The volcano plots displayed 195 down-regulated circRNAs and 167 up-regulated circRNAs (P values < 0.05, |log2(FC)| >1)

First, in the screening analysis, we investigated the key circRNAs in SLE. Total RNA was extracted from the peripheral blood of 4 SLE patients and 4 healthy volunteers, which showed no differences in sex or age (P > 0.05). High-throughput RNA-Seq was conducted based on the digested-RNA with Rnase R. The data matrix from RNA-Seq was collected and analyzed using R software. According to the standard of “P values < 0.05, | log2 (FC) | > 1”, 362 differentially dysregulated circRNAs between SLE patient and healthy volunteers were screened. The volcano plot and heatmap analysis revealed that 195 circRNA were significantly down-regulated and 167 were significantly up-regulated in SLE (Fig. 1).

### Enrichment analyses

**Fig. 2:**
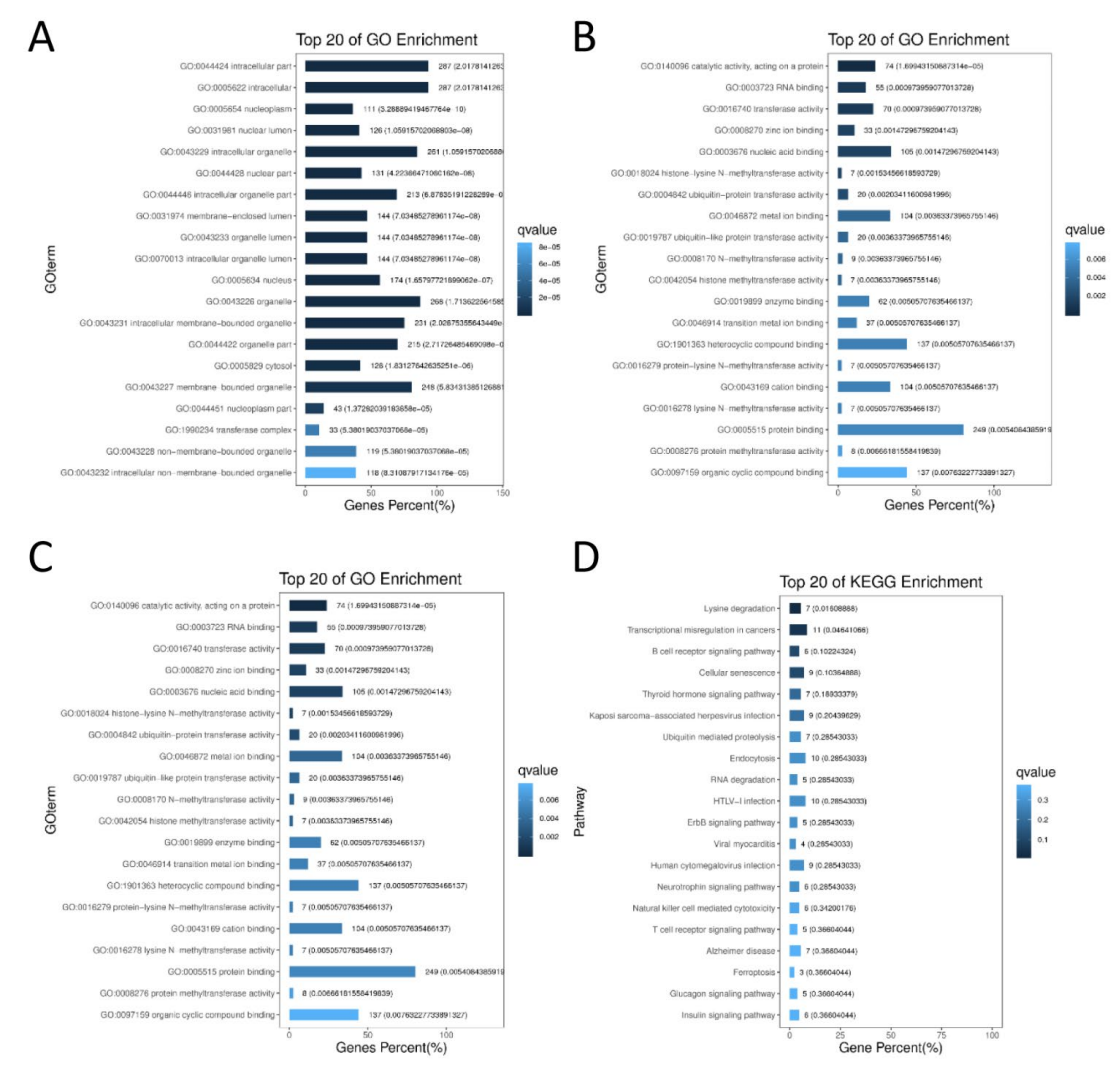
Functional and pathway enrichment analyses from differentially expressed circRNAs in SLE patients. (A) Biological process GO analysis; (B) cell component GO analysis; and (C) molecular function GO analysis of differentially expressed circRNAs in SLE. (D) KEGG enrichment analysis of differentially expressed circRNAs in SLE.

GO analysis of circRNA source genes that were differentially expressed between the SLE and healthy control groups included three categories: biological process, cell component, and molecular function. Biological process analysis showed that the source genes were involved in cellular macromolecule metabolic processes, organelle organization, macromolecule modification, cellular protein modification processes, protein modification processes, cellular metabolic processes, cellular protein metabolic processes, macromolecule metabolic processes, chromatin organization, and regulation of cellular metabolic processes. The results suggested circRNAs differentially expressed in PBMCs of SLE patients were highly related to cell metabolism and molecular modifications. Cell component analysis indicated that the genes regulated by the differentially expressed circRNAs were mainly distributed across the categories of intracellular part, intracellular, nucleoplasm, nuclear lumen, intracellular organelle, nuclear part, intracellular organelle part, membrane-enclosed lumen, organelle lumen, and intracellular organelle lumen, indicating that these genes were mostly enriched in the cytoplasm. Molecular function analysis implied the circRNA-regulated genes were mainly enriched in catalytic activity, acting on proteins, RNA binding, transferase activity, zinc ion binding, nucleic acid binding, histone-lysine N-methyltransferase activity, ubiquitin-protein transferase activity, metal ion binding, ubiquitin-like protein transferase activity, and N-methyltransferase activity (Fig. 2).

KEGG pathway enrichment analysis identified the pathways of lysine degradation, transcriptional mis-regulation in cancers, B cell receptor signaling, cellular senescence, thyroid hormone signaling, Kaposi sarcoma-associated herpesvirus infection, ubiquitin-mediated proteolysis, endocytosis, RNA degradation, HTLV-l infection, ErbB signaling, viral myocarditis, human cytomegalovirus infection, neurotrophin signaling, natural killer cell-mediated cytotoxicity, and T cell receptor signaling as key pathways influenced by the differentially expressed circRNAs in SLE.

### Validation of the differentially expressed circRNAs in PBMCs from SLE patients and healthy controls

To further identify the key circRNAs that participate in the process of SLE development and progression, a false discovery rate (FDR) < 0.2 was used to further narrow the range of candidate circRNAs and identify circRNAs with high sequencing accuracy among the previously screened differentially expressed circRNAs. We selected the following circRNAs with large differences in expression: hsa_circ_0006689, hsa_circ_0070562, hsa_circ_0006117, hsa_circ_0007683, hsa_circ_0042519, and hsa_circ_0008647.

**Fig. 3:**
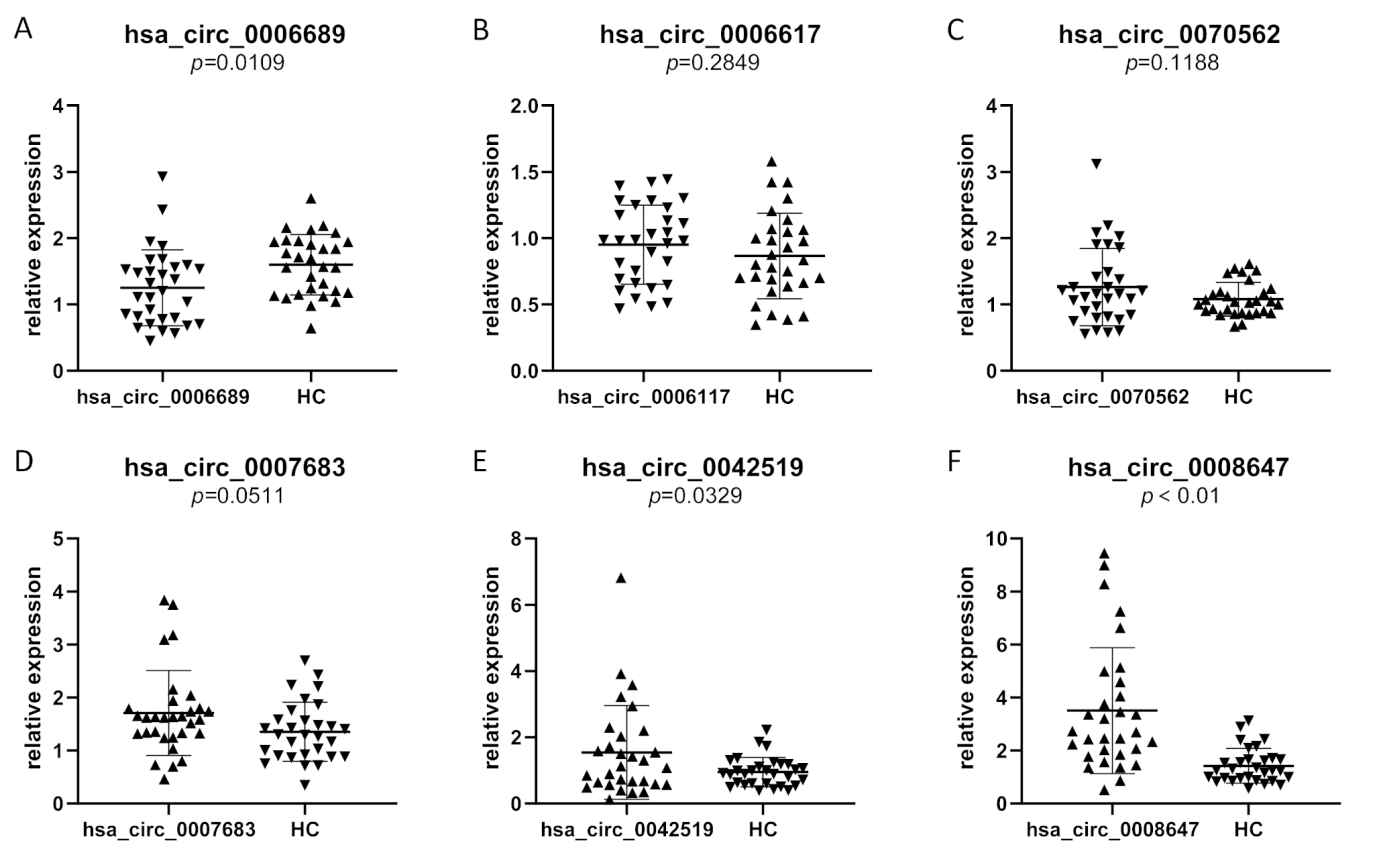
qPRC validation of the differential expression of circRNAs in PBMCs from SLE patients and healthy controls (HC). (A-D) Relative expression levels of differentially expressed circRNAs in SLE patients compared with healthy controls as determined by qPCR.

Then, we designed experiments to confirm the expression levels of these circRNAs. A validation experiment to confirm the differential expression of these circRNAs in PBMCs by qPCR was conducted using samples from 31 SLE patients and 35 healthy volunteers (healthy controls). Relative expression levels of the circRNAs were obtained by setting the median levels in healthy controls as 1. As shown in Fig. 3, the relative expression levels of hsa_circ_0042519, and hsa_circ_0008647 were significantly higher in the SLE group than healthy control group (P < 0.05). Conversely, hsa_circ_0006689 was down-regulated in the SLE group (P = 0.0109). The expression levels of hsa_circ_0070562, hsa_circ_0006117 and hsa_circ_0007683 showed no significant different between the SLE and healthy control groups.

### Correlations between differential circRNA expression and clinical variables in SLE patients

**Fig. 4:**
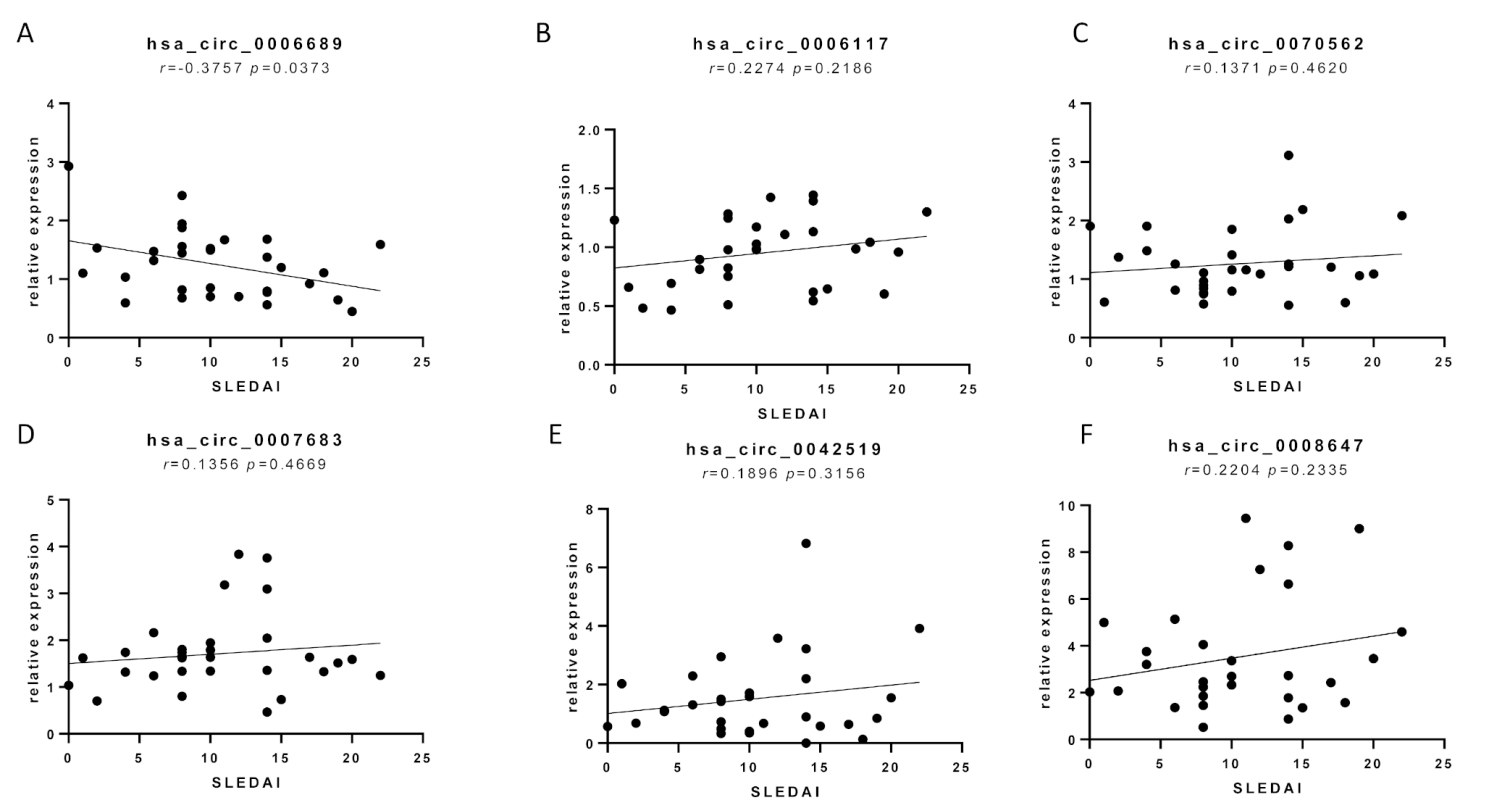
Correlation of circRNAs expression and SLEDAI score. (A-F) Spearman correlation analysis showed that only the expression level of hsa_circ_0006689 in PBMCs was correlated with the SLEDAI score (P < 0.05)

The SLEDAI scoring system is widely used to assess disease activity in SLE patients (Gladman et al. 2002). We collected the SLEDAI scores for the included SLE patients and investigated correlations between circRNAs expression levels and the SLEDAI score. Spearman correlation analysis was performed for the expression levels of hsa_circ_0007683, hsa_circ_0008647, hsa_circ_0042519, and hsa_circ_0006689, which were verified as differentially expressed by qRT-PCR. The results indicated the expression level of hsa_circ_0006689 in PBMCs correlated with the SLEDAI score (P < 0.05), whereas the expression levels of hsa_circ_0007683, hsa_circ_0042519, and hsa_circ_0008647 showed no correlation with the SLEDAI score (P > 0.05; Fig. 4). These findings indicate that hsa_circ_0006689 may be a useful prognostic marker in SLE.

### Potential diagnostic value of differentially expressed circRNAs among SLE patients

**Fig. 5:**
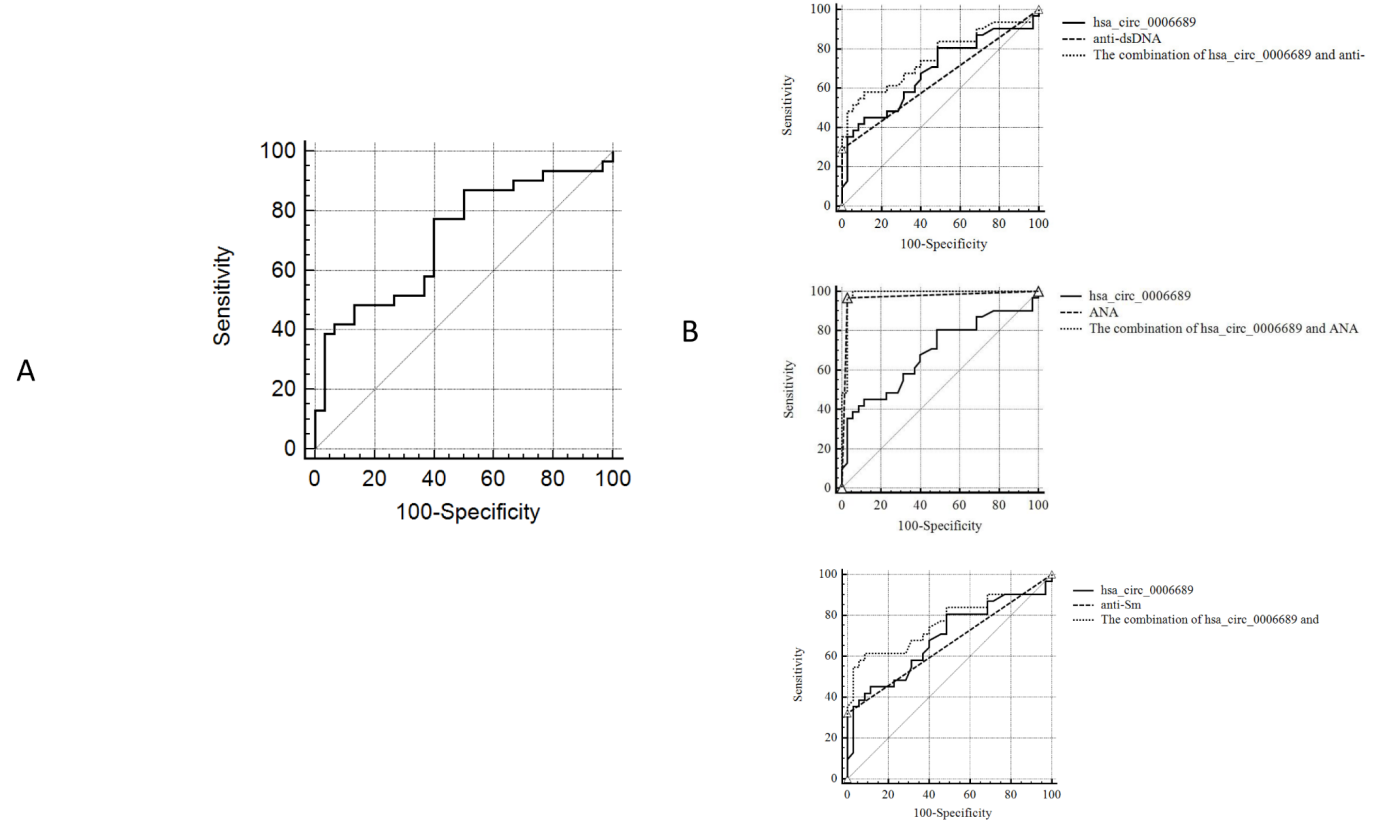
Sensitivity and specificity of different biomarkers for the diagnosis of SLE. ROC curve analysis was conducted, and AUC values were determined. (A) ROC curve analysis of hsa_circ_0006689 in the SLE and healthy control groups. (B) ROC curve analysis for the combination of hsa_circ_0006689 and other current diagnostic biomarkers in the SLE and healthy control groups

Based on the findings that hsa_circ_0006689 expression may be a valuable biomarker in SLE, ROC curve analysis was performed to evaluate the diagnostic performance of hsa_circ_0006689 expression for SLE. The area under the ROC curve (AUC) was found to be 0.713 (95% confidence interval: 0.5824–0.8434; P < 0.001) with a sensitivity of 51.61% and specificity of 88.57% (Fig. 5 A).

Anti-dsDNA antibodies, ANAs, ANuA, and anti-Sm antibody are the most commonly used markers for SLE (Yu et al. 2014). Thus, we further explored whether any diagnostic improvement can be gained by adding hsa_circ_0006689 expression to these markers. ROC curve analysis of the ability hsa_circ_0006689 in addition to ANA, anti-dsDNA antibody, or anti-Sm antibody to identify SLE was conducted. Combined detection of hsa_circ_0006689 with anti-dsDNA antibody and anti-Sm antibody in the healthy control group and SLE group showed an increase in sensitivity from 29.03 to 61.30% compared with anti-dsDNA antibody only and from 32.26 to 71.00% compared with anti-Sm antibody only (Fig. 5B). These differences in sensitivity with and without the addition of hsa_circ_0006689 were statistically significant (P < 0.05). Our results suggest the strong potential of hsa_circ_0006689 expression as a biomarker for the diagnosis of SLE.

## Discussion

Due to the complexity of SLE pathogenesis, a single efficient biomarker for the diagnosis and prognosis of SLE has yet to be identified. In the present study, RNA-Seq was performed with peripheral blood samples from SLE patients and healthy individuals to identify circRNAs that are differentially expressed in SLE. The circRNAs identified in this screening analysis were further examined to determine the key target in SLE. Through validation by qPCR, evaluation of correlations with SLEDAI score, and ROC curve analysis, our study found that hsa_circ_0006689 could be a potent prognostic and diagnostic marker for SLE. Consistent with the above results, our data indicate that hsa_circ_0006689 can serve as a useful biomarker for diagnosis and prognosis evaluation in SLE patients. Our results provide new ideas and strategies for the diagnosis and treatment of SLE.

Because circRNA expression is very stable in vivo, these RNAs can provide good biomarkers. Recently, some circRNAs found in PBMCs and plasma were reported as potential biomarkers for SLE. For example, the expression of circIBTK was down-regulated and the expression of miR-29b was up-regulated in PBMCs of SLE patients, and the expression of both circRNAs correlated with the level of anti-dsDNA antibodies and SLEDAI score (Wang et al. 2018). Another study reported that the expression levels of hsa_circ_0049224 and hsa_circ_0049220 were decreased in PBMCs of patients. Moreover, the expression levels of these two circRNAs in PBMCs were negatively correlated with the SLEDAI score, suggesting their potential role as biomarkers for the evaluation of SLE activity (Zhang et al. 2018). Additionally, in SLE patients with lupus nephritis (LN), hsa_circRNA_002453 expression in plasma is higher than that in SLE patients with no lupus nephritis. Further, they reported that the hsa_circRNA_002453 expression level is positively correlated with the SLEDAI score and 24-hour urine albumin level. These data imply hsa_circRNA_002453 has potential value in the assessment of renal damage in SLE patients (Ouyang et al. 2018). Together, these results provided evidence that circRNAs have the potential to be new diagnostic markers and indicators of disease activity in SLE. However, most of the current research profiled circRNA biomarkers of SLE by microarray analysis, while high-throughput sequencing offers the potential to discover novel circRNAs and higher accuracy. Moreover, most of subjects of those studies were not previously untreated patients, and this might affect the profiling of circRNAs. In our study, hsa_circ_0006689 was not found in the public data for circRNA profiling in SLE, and the other circRNAs reported were not validated in our circRNA profile.

In the present study, we screened a total of 362 circRNAs with significant differences in expression between SLE patients and healthy volunteers through high-throughput RNA-Seq analysis. Compared to relevant studies using circRNA microarrays, the number of differentially expressed circRNAs obtained by high-throughput sequencing was considerably greater (Su et al. 2019). Additionally, we used KEGG enrichment analysis to identify enriched pathways related to the differentially expressed circRNA source genes and found that the enrichment of target genes was mainly related to lysine degradation, tumor error transcription, B cell receptor signaling, and other pathways (Fig. 2). There have been many reports on the correlations between SLE and tumor immunity and B-cell signal recognition pathways, and our pathway analysis suggested that the differentially expressed circRNA source genes were enriched in the lysine-related pathway, which interested us. Lysine does not appear to be associated with SLE but is necessary to assist T cells in the specific recognition of antigens. Lysine has been reported to be a specific “bridging” component that binds antigens to T cells and promotes the production of specific immunity (Li et al. 2007). When lysine is lacking in the body, lymphocyte proliferation is inhibited and the cellular immune response is affected (Ghosh et al. 2008).

Hsa_circ_0006689 is derived from the gene for SLC15A4 (Solute Carrier family 15, member 4), which belongs to the proton-coupled histone and oligopeptide transporter family. In 2009, two single nucleotide polymorphisms (rs10847997 and rs1385374) of SLC15A4 were first identified in a Chinese Han cohort (Han et al. 2009). Subsequently, GWAS further showed that *SLC15A4* is a susceptibility gene for SLE, and its SNP mutation plays a key role in the pathogenesis of SLE (Lee et al. 2014; Zhang et al. 2016). Studies have shown that SLC15A4 promotes the production of cytokines such as interferon and nuclear factor-κB in dendritic cells, B cells and other immune cells by activating Toll-like receptor, NOD and other signaling pathways and thereby plays an important role in the occurrence and development of SLE (Griffith et al. 2018). In our study, we found that hsa_circ_0006689 was significantly down-regulated in SLE patients. Combined with previous studies, our study suggests that hsa_circ_0006689, which is derived from *SLC15A4*, may play an important role in the occurrence and development of SLE, further confirming the potential of hsa_circ_0006689 as a biomarker for SLE.

Our study has several limitations. First, the sample size was small, with only 4 SLE patients providing samples for the RNA-Seq experiment. Given the huge differences among human tissue samples, the amount of 12 paired samples is considered a lower limitation generally. Due to funding and informed consent issues, we could not enroll enough subjects in our study to reach this sample size. Secondly, we used samples from healthy people as the internal control for SLE patients without comparing circRNA expression in patients with other autoimmune diseases. This study design was not rigorous enough, as circRNA expression could also be regulated by different immune states of the body. In addition, our study only included a general analysis of all types of SLE, even though SLE is a complex disease with diverse subtypes. Future research is needed to analyze the differential cicrRNA expression profiles of various subtypes of SLE.

Despite its limitations, our study first identified differentially expressed circRNAs between 4 SLE patients and 4 healthy volunteers. Then qPCR, Spearman correlation analysis and ROC curve analysis verified hsa_circ_0006689 as a potentially useful circRNAs biomarker for SLE diagnosis and prognosis evaluation. Thus, our study provides a new biomarker and diagnostic approach for SLE. Our findings provide valuable insight for the clinical diagnosis, treatment, and mechanism of SLE.

## Data Availability

The data are available from the corresponding author on reasonable request.

## Declarations

This work was supported by the National Natural Science Foundation of China (81760561); Natural Science Foundation of Guangxi Province (2020GXNSFAA297150).
